# Impact of implementing a free varicella vaccination policy on incidence in Wuxi City, China: an interrupted time series analysis

**DOI:** 10.1017/S0950268823001152

**Published:** 2023-07-30

**Authors:** Shixin Xiu, Xuwen Wang, Qiang Wang, Hui Jin, Yuan Shen

**Affiliations:** 1 Department of Immunization, Wuxi Center for Disease Control and Prevention, Wuxi, China; 2 Department of Epidemiology and Health Statistics, School of Public Health, Southeast University, Nanjing, China; 3 Key Laboratory of Environmental Medicine Engineering, Ministry of Education, School of Public Health, Southeast University, Nanjing, China

**Keywords:** children, expanded program of immunisation, incidence, interrupt time series, Varicella vaccine

## Abstract

Varicella vaccination is optional and requires self-payment. On 1 December 2018, Wuxi City launched a free varicella vaccination program for children. This study aimed to evaluate the changes in varicella incidence before and after the implementation of the policy. The data were obtained from official information systems and statistical yearbooks. We divided the period into chargeable (January 2017 to November 2018) and free (December 2018 to December 2021) periods. Interrupt time series analysis was used to conduct a generalised least-squares regression analysis for the two periods. A total of 51,071 varicella cases were reported between January 2017 and December 2021. After the implementation of the policy, there was a statistically significant decrease in the incidence of varicella (β2 = −0.140, P = 0.017), and the slope of the incidence also decreased by 0.012 (P = 0.015). Following policy implementation, the incidence decreased in all age groups, with the largest decline observed among children aged 8–14 years (β2 = −1.109, P = 0.009), followed by children aged ≤7 years (β2 = −0.894, P = 0.013). Our study found a significant reduction in the incidence of varicella in the total population after the introduction of free varicella vaccination in Wuxi City.

## Introduction

Varicella is an acute infectious disease caused by varicella–zoster virus [1]. The World Health Organization (WHO) estimates that it causes approximately 140 million cases every year globally, leading to four million severe complications requiring hospitalisation and 4,200 deaths [2]. Studies have demonstrated the clinical effectiveness of the varicella vaccine, and some high-income countries such as the United States, Italy, Sweden, and the United Kingdom have reported its cost-effectiveness [3–7]. As of 2019, the WHO reported that varicella vaccination was part of the routine childhood immunisation programs in 50 of 194 member states [8].

Varicella is the third most common vaccine-preventable infectious disease in mainland China, with an estimated more than three million new cases in 2019, following tuberculosis and influenza [9]. China first introduced the varicella vaccine in 1998 [10]. A meta-analysis estimated that three-fifths of children in China received the varicella vaccination [11]. The varicella vaccine is not included in China’s National Expanded Program on Immunization (EPI), which means it is an optional vaccine that requires self-payment [12]. Currently, several studies have indicated that free varicella vaccination for children could be a cost-effective strategy in China [9, 13]. To mitigate the impact of the varicella-related burden, some provinces and cities in China, such as Shanghai and Nanjing, have added varicella to their immunisation programs and offer free varicella vaccination to children [9]. The effectiveness of implementing a free vaccination policy to reduce the incidence of varicella has been infrequently studied. Gao et al. [14] found that the varicella incidence decreased after introducing the free varicella vaccination policy. Further analysis is needed to examine the impact of the free varicella vaccination policy on different age groups and to consider the potential seasonality of varicella incidence.

On 1 December 2018, Wuxi City in eastern China launched a free varicella vaccination program for all preschool children. To support evidence-based health decision-making, it is essential to understand varicella vaccination coverage and to evaluate the incidence of varicella in Wuxi City. This study aimed to evaluate the changes in the incidence of varicella before and after the introduction of varicella vaccination into the EPI and examined its patterns at various levels (e.g. age groups).

## Methods

### Data sources

Wuxi is situated in eastern China, south of the Jiangsu Province, and has a population of more than 7 million and an area of 4,627 square kilometres (Supplementary Figure S1). We collected varicella case information from the China Disease Control and Prevention Information System from 1 January 2017 to 31 December 2021 including data on birth dates, sex, and dates of onset of varicella cases. Varicella vaccination data were obtained from the Wuxi Comprehensive Vaccination Service Management Information System, including the target population size and the actual number of people administrated the vaccine. Additionally, annual age-specific population data were derived from the Wuxi statistical yearbook [15].

### Varicella vaccination policy

In Wuxi City, before December 2018, varicella vaccination was categorised as a non-EPI vaccine and was not mandatory. Individuals were required to pay for the vaccine upon receiving it. After 1 December 2018, free varicella vaccination became available for children with no history of varicella infection. This policy included a routine and emergency vaccination program. Children aged <12 months received the first dose at 12 months and the second dose at 48 months. A routine catch-up vaccination strategy has also been implemented. Children aged 12–48 months received the first dose as soon as possible and the second dose at 48 months. Children aged 48–84 months (7 years) received two doses. It is mandatory for vaccinated individuals to complete both doses of the vaccine by the age of 7 years. Furthermore, the varicella vaccine has also been utilised as post-exposure prophylaxis during varicella outbreaks to control viral transmission [16]. In the case of varicella outbreaks in kindergartens, primary schools, and secondary schools, close contacts of varicella cases who have no infection history or vaccination experience need to be promptly vaccinated as an emergency vaccination program.

### Data analysis

This study describes vaccination coverage and varicella cases, with qualitative data expressed as frequency (%) and quantitative data as mean ± standard deviation. We divided the period into non-EPI (January 2017 to November 2018) and EPI (December 2018 to December 2021) periods, with the policy implementation node as the boundary. Interrupt time series (ITS) analysis was used to conduct generalised least-squares regression analysis for the two time periods before and after the introduction of the varicella vaccine into planned immunisations, analysing changes in the level and slope of varicella-related morbidity caused by the policy [17]. The regression model is as follows:

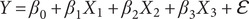



where Y is the incidence of varicella (per 1,000 person months). *X*_1_ denotes time, and the value was one, two, three… 60, representing each month from January 2017 to December 2021. *X*_2_ represents intervention, which reflected the introduction of varicella vaccine into the EPI. The policy began in December 2018, and the value was zero before the implementation of the policy and one after implementing the policy. Postslope is denoted as *X*_3_ and was set to zero before the implementation of the policy and ranged from 1 to 36 after the implementation. β_1_ denotes the slope before the introduction of free vaccination; β_2_ denotes the level change which could show the immediate effect of varicella vaccination; β_3_ represents the slope which could show the long-term effect of varicella vaccination; Ɛ is error; and β_0_ is the intercept. The Durbin–Watson (DW) method was used to determine whether there was evidence of autocorrelation [18]. The DW statistic has a range from 0 to 4, and values near 2 suggest the absence of autocorrelation. The Prais–Winsten method was used to adjust the results if the Durbin–Watson statistic, indicating the presence of autocorrelation (far from two) [19].

A sensitivity analysis was performed. We examined the differences between the effect of the routine and emergency vaccination programs on decreasing varicella incidence by age stratification, namely, children aged ≤7 years (population for routine vaccination), children aged 8–14 years (population for emergency vaccination), and individuals aged >14 years. Considering the impact of the COVID-19 pandemic (a potentially low rate of reporting cases), we also analysed the changes in varicella incidence during the pre-pandemic period (from January 2017 to December 2019). Additionally, because the incidence of varicella tends to show a seasonal pattern, we conducted an analysis to adjust for seasonality. This involved decomposing the fluctuation trend into a long-term trend, periodic trend (seasonal), and random change, and subtracting the initial incidence from the periodic trend to obtain the incidence of varicella without seasonality.

Data analysis was performed using R software, and all statistical tests were two-sided, with a significance level of α = 0.05.

## Results

**Figure 1.**
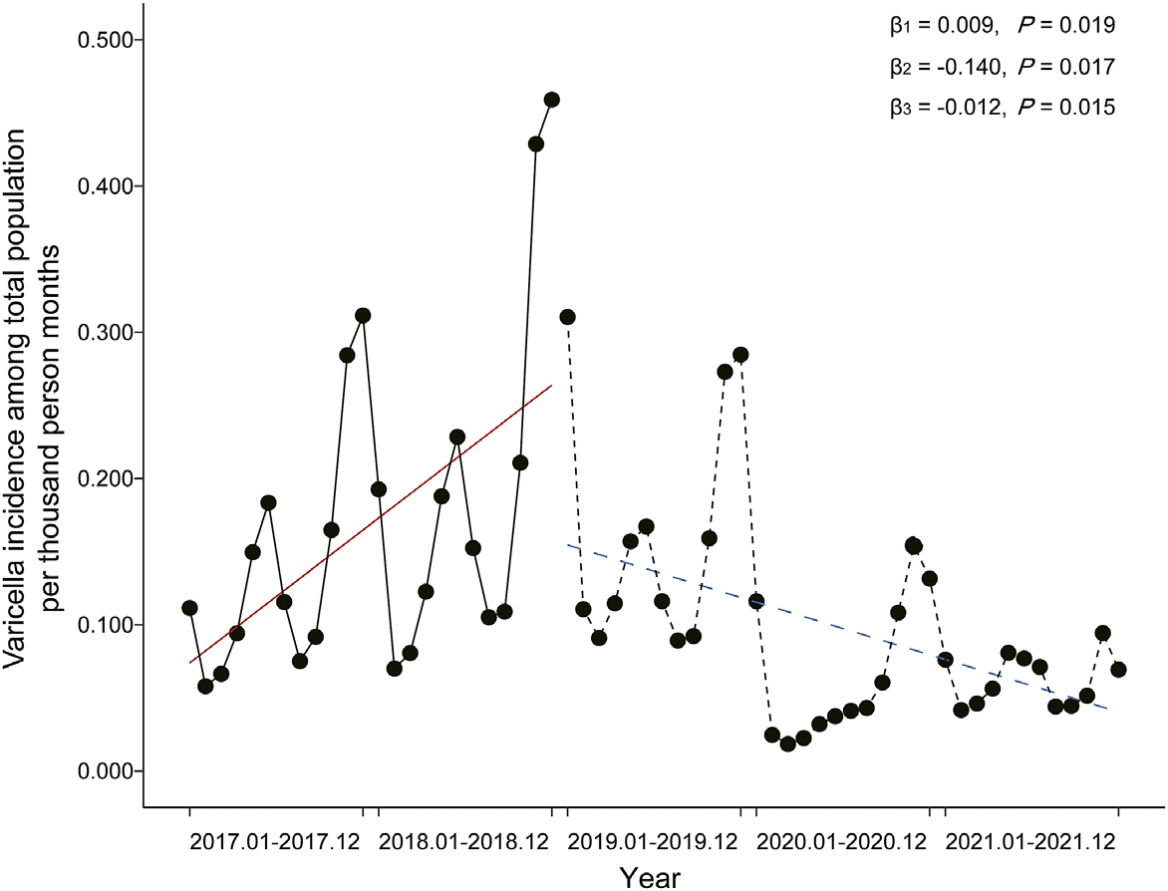
Varicella incidence among the total population in Wuxi City from 2017 to 2021

A total of 51,071 varicella cases were reported in Wuxi City between January 2017 and December 2021, with an average age of 12.927 ± 10.092 years (Table 1). Among these cases, the age group of individuals aged 8–14 years accounted for the largest proportion. Over a 5-year period, the incidence of varicella ranged from 0.001 to 0.116 per thousand individuals per month. The incidence showed a declining trend from January to March and from June to August each year, followed by an increasing trend starting in September (Figure 1 and Supplementary Figure S2). From 2017 to 2021, the percentage of individuals who received the first dose of the varicella vaccine increased from <60% to >97%, whereas the percentage of those who received the second dose increased from <5% to >91% (Table 2).

**Table 1.** Characteristics of varicella cases in Wuxi from 2017 to 2021

Variable		Frequency(%)
Age	≤7 years old	16,130(31.583)
	8–14 years old	18,761(36.735)
	>14 years old	16,180(31.681)
Gender	Male	27,281(53.418)
	Female	23,790(46.582)
Year	2017	11,178(21.887)
	2018	15,430(30.213)
	2019	12,952(25.361)
	2020	5,894(11.541)
	2021	5,617(10.998)
Total		51,071

**Table 2.** Varicella vaccination rates from 2017 to 2021

Year	First dose				Second dose		
	Target population size	Actual number of people administrated vaccine	Coverage rate(%)		Target population size	Actual number of people administrated vaccine	Coverage rate(%)
2017	444,335	264,659	59.563		151,054	6,485	4.293
2018	451,678	287,950	63.751		162,575	18,465	11.358
2019	432,873	365,694	84.481		168,928	67,180	39.768
2020	423,584	384,507	90.775		198,380	149,436	75.328
2021	390,191	379,239	97.193		210,916	192,037	91.049

**Figure 2.**
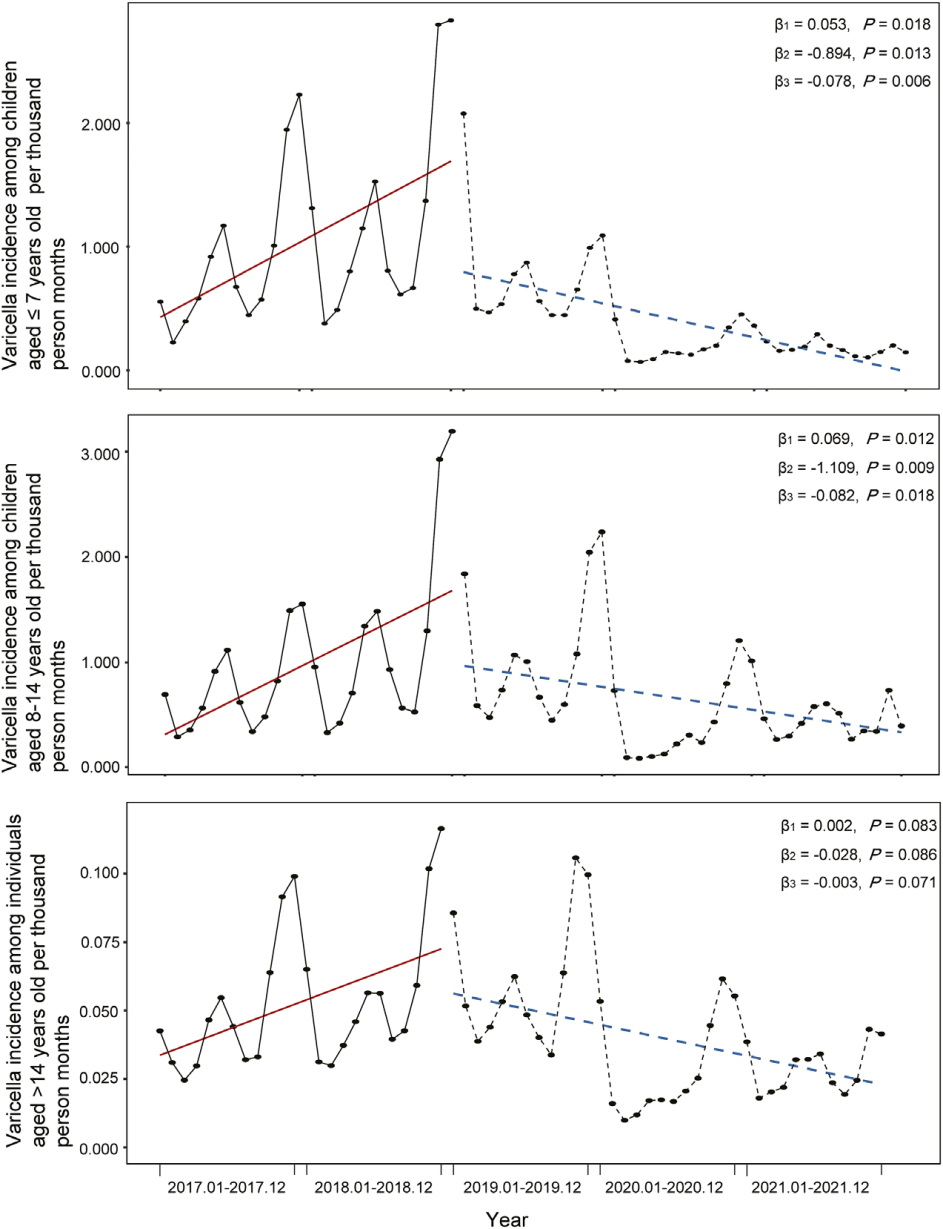
Varicella incidence among three age groups in Wuxi City from 2017 to 2021

The DW statistic indicated that there might be autocorrelation, and we employed the Prais–Winsten method in the analyses (Supplementary Table S1). During the non-EPI period, there was a statistically significant increase in the incidence of varicella in the total population, with an increase of 0.009 per thousand person months (*P* = 0.019; Figure 2). After the vaccine was introduced into the EPI, there was a statistically significant decrease in the incidence of varicella, with a reduction of 0.140 per thousand person months compared with the non-EPI period (*P* = 0.017). The slope of incidence also decreased by 0.012 (*P* = 0.015) after the EPI, which indicated a decrease in incidence by 0.003 (β_1_ + β_3_:0.009–0.012) per thousand person months after the policy was implemented.

Before policy implementation, the incidence of varicella was increased in all three age groups (Figure 2). Following policy implementation, the incidence decreased in all three age groups, with the largest decline observed among children aged 8–14 years (β_2_ = −1.109, *P* = 0.009), followed by children aged ≤7 years (β_2_ = −0.894, *P* = 0.013). The slope of incidence among children aged ≤7 years decreased by −0.078 after EPI implementation (*P* = 0.006), indicating that the incidence decreased by 0.025 per thousand person months after policy implementation.

During the pre-pandemic period (Supplementary Table S2), there was a slight upward trend in the varicella incidence among the total population, with an increase of 0.009 per thousand person months; however, this difference was not statistically significant (*P* = 0.065). After the introduction of the varicella vaccine into the EPI, there was a significant reduction in varicella incidence by 0.149 per thousand person months compared with the non-EPI period (*P* = 0.049). Among children aged 8–14 years, there was a significant reduction in varicella incidence by 1.296 per thousand person months compared to the non-EPI period (*P* = 0.015). There was no statistically significant difference in the slope of varicella incidence (β_3_) among children aged ≤7 years (*P* = 0.338), children aged 8–14 years (*P* = 0.662), and individuals aged >14 years (*P* = 0.644). These results suggest that the EPI did not have a long-term effect on varicella incidence in these three age groups prior to the COVID-19 pandemic.

After removing the seasonal effect (Supplementary Figure S3), the incidence of varicella in the total population increased significantly before the implementation of the policy (β_1_ = 0.006, *P* = 0.017). During the EPI period, the varicella incidence decreased by 0.037 per thousand person months compared to the non-EPI period, but the difference was not statistically significant (*P* = 0.250). The slope of the incidence among the total population decreased by −0.012 (*P* < 0.001) after the EPI, indicating that the incidence decreased by 0.006 (β_1_ + β_3_:0.006–0.012) per thousand person months after the implementation of the policy. The varicella incidence among children aged ≤7 years, children aged 8–14 years, and individuals aged >14 years decreased by 0.041, 0.038, and 0.002 per thousand person months, respectively, after policy implementation, and the EPI had long-term effects on the varicella incidence among all three age groups.

## Discussion

Our study revealed a significant reduction in the varicella incidence after the introduction of varicella vaccination into the EPI in Wuxi City, which is consistent with the findings of other studies [10, 20, 21]. This decrease was observed across all age groups, including children aged ≤7 years, children aged 8–14 years, and individuals aged >14 years. Notably, the most significant decreases were observed among children aged ≤7 years and children aged 8–14 years.

Since 2020, COVID-19 prevention measures, such as wearing masks and lockdown policies, have become confounding factors that affect the incidence and reporting of varicella cases in the population. However, the sensitivity analysis results showed a significant decline in the varicella incidence in the 13 months between policy implementation and the COVID-19 outbreak (from December 2018 to December 2019). Therefore, vaccination may play a crucial role in reducing the incidence of varicella in the population. Even after eliminating seasonal effects, it is clear that the inclusion of the varicella vaccine in the EPI led to a significantly decreasing incidence trend compared with that before inclusion.

It is worth noting that the age group of 8–14 years showed a greater decline in morbidity than the age group of 7 years and below, indicating the potentially critical role of emergency immunisation, which is consistent with other studies [22, 23]. It is possible that the target population for emergency immunisation is at a higher risk of infection and has a history of immunisation, which may explain the more pronounced effects of emergency immunisation.

Our findings demonstrated a decrease in varicella incidence from January to March and from June to August, which aligns with previous epidemiological studies conducted in various regions of China, including Shandong Province, Dalian City, Guangzhou City, and Hangzhou City [24–27]. Cheng et al. and Lu et al. suggested that this period corresponds to the winter and summer vacations of Chinese students [25, 26]. The occurrence of summer and winter holidays in many schools across China during this timeframe led to decreased interactions among children, thereby reducing the likelihood of varicella transmission within educational environments where the virus can easily spread. The literature has shown that a one-dose varicella vaccine is more cost-effective than a two-dose vaccine [28], but the rate of breakthrough infections with a one-dose vaccine is higher than that with a two-dose vaccine [29, 30]. Therefore, considering multiple perspectives of evidence, it is essential to provide children with two doses of the routine varicella vaccine, and high coverage of two-dose varicella vaccination should be achieved to maximise the benefits of the varicella vaccination program [31, 32].

## Limitations

Our study had a few limitations. First, the analysis only covered a relatively short period because Jiangsu Province has only been monitoring and reporting the number of level C infectious diseases, with varicella as a reference, since 2017. The full effect of this policy may not become evident until more data are collected. Second, the COVID-19 pandemic has affected the management and reporting of infectious diseases [33, 34], and social measures implemented to prevent COVID-19 may also affect the incidence of varicella [35]. Despite conducting sensitivity analyses, the effectiveness of the vaccine could be compromised by the short timeframe.

## Conclusion

Our study found a significant reduction in the incidence of varicella in the total population after the introduction of varicella vaccination into the EPI in Wuxi City in December 2018. This decrease was observed across all age groups, including children aged ≤7 years, children aged 8–14 years, and individuals aged >14 years. After removing the impact of the COVID-19 pandemic and seasonality on incidence, the pattern of decrease could also be observed among children aged ≤7 years and children aged 8–14 years.

## Supporting information

Xiu et al. supplementary material Xiu et al. supplementary material

## Data Availability

Data available within the article or its supplementary materials.

## Supplementary material

For supplementary material accompanying this paper visit http://doi.org/10.1017/S0950268823001152. click here to view supplementary material
