# Supplementary material for: Impact of implementing a free varicella vaccination policy on incidence in Wuxi City, China: an interrupted time series analysis
Source: Epidemiol Infect. 2023 Jul 30;151:e125. doi: 10.1017/S0950268823001152 (PMC10540171; doi:10.1017/S0950268823001152)
Supplement: Xiu et al. supplementary material [file S0950268823001152sup001.docx]

***Epidemiology and Infection***

**Impact of implementing free varicella vaccination policy on incidence in Wuxi city, China: An interrupted time-series analysis**

**Shixin Xiu, Xuwen Wang, Qiang Wang, Hui Jin, Yuan Shen**

*Supplementary Material*

Figure S1. Wuxi city in China

Figure S2. Varicella incidence and vaccination coverage

Table S1. Results of autocorrelation

Table S2. Effect of the inclusion of varicella vaccine in the EPI on varicella incidence

Figure S3. Decomposing of varicella incidence from 2017 to 2021


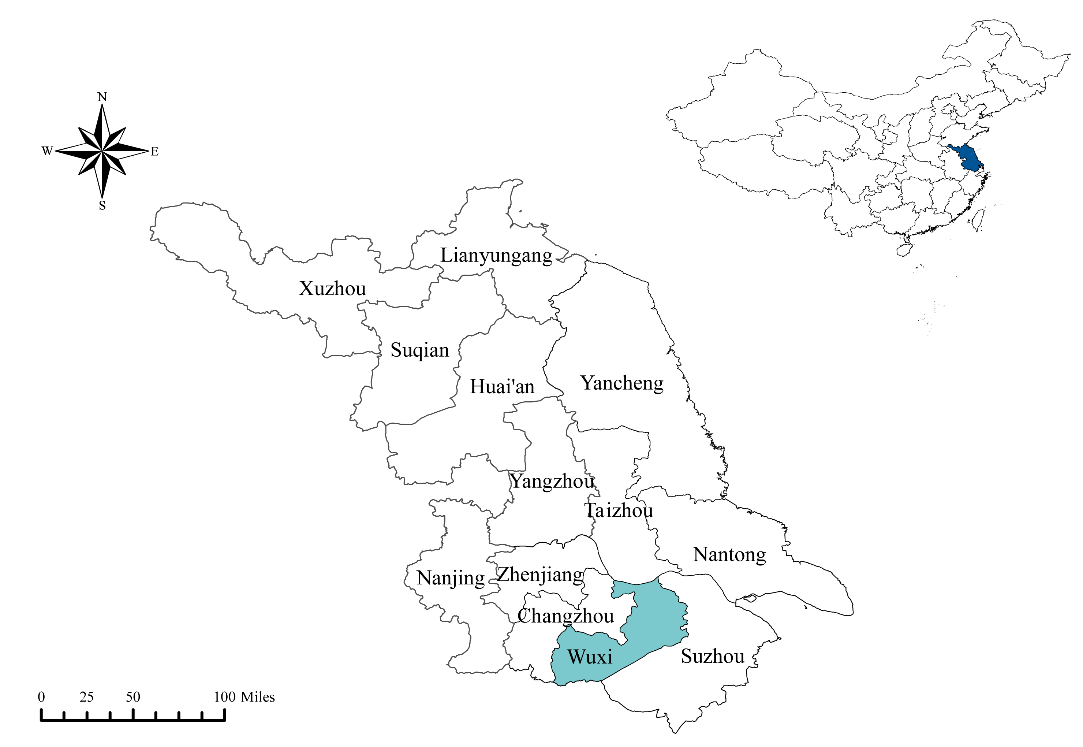


**Figure S1. Wuxi city in China**


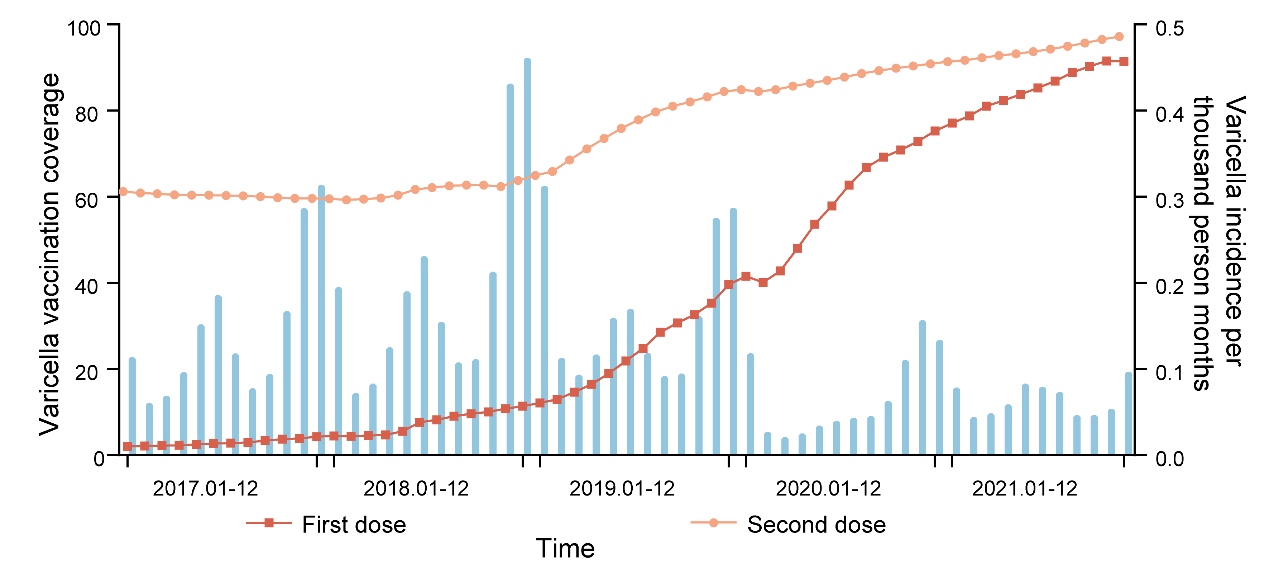


**Figure S2. Varicella incidence and vaccination coverage^#^**

^#^ Coverage by month was calculated by dividing the number of children aged 12 months to 84 months (eligible for varicella vaccination) getting vaccination by the total age-specific number of children at a given month.

**Table S1. Results of autocorrelation**

| Modelling | | Original D-W statistic | Transformed D-W statistic after prais-Winsten |
| --- | --- | --- | --- |
| Total population | | 0.757 | 1.139 |
| Age | ≤7 years old | 0.838 | 1.330 |
|  | 8-14 years old | 0.788 | 1.159 |
|  | >14 years old | 0.657 | 1.003 |
| Before the COVID-19 pandemic | Total population | 0.768 | 1.150 |
|  | ≤7 years old | 0.858 | 1.308 |
|  | 8-14 years old | 0.743 | 1.118 |
|  | >14 years old | 0.709 | 1.083 |
| Excluding seasonality | Total population | 0.602 | 1.183 |
|  | ≤7 years old | 0.757 | 1.398 |
|  | 8-14 years old | 0.588 | 1.141 |
|  | >14 years old | 0.569 | 1.460 |

**Table S2. Effect of the inclusion of varicella vaccine in the EPI on varicella incidence**

| Subgroup | | Variable | $\beta$-value | t-value | *P-*value |
| --- | --- | --- | --- | --- | --- |
| Before the COVID-19 pandemic | Total population | β_1_ | 0.009 | 1.908 | 0.065 |
|  |  | β_2_ | -0.149 | -2.044 | 0.049 |
|  |  | β_3_ | -0.001 | -0.101 | 0.920 |
|  | ≤7 years old | β_1_ | 0.056 | 1.975 | 0.057 |
|  |  | β_2_ | -0.815 | -1.668 | 0.105 |
|  |  | β_3_ | -0.077 | -0.974 | 0.338 |
|  | 8-14 years old | β_1_ | 0.063 | 1.970 | 0.058 |
|  |  | β_2_ | -1.296 | -2.570 | 0.015 |
|  |  | β_3_ | 0.039 | 0.442 | 0.662 |
|  | >14 years old | β_1_ | 0.002 | 1.483 | 0.148 |
|  |  | β_2_ | -0.031 | -1.703 | 0.098 |
|  |  | β_3_ | 0.002 | 0.467 | 0.644 |
| Excluding seasonality | Total population | β_1_ | 0.006 | 2.459 | 0.017 |
|  |  | β_2_ | -0.037 | -1.162 | 0.250 |
|  |  | β_3_ | -0.012 | -3.740 | <0.001 |
|  | ≤7 years old | β_1_ | 0.037 | 2.244 | 0.029 |
|  |  | β_2_ | -0.358 | -1.457 | 0.151 |
|  |  | β_3_ | -0.078 | -3.613 | 0.001 |
|  | 8-14 years old | β_1_ | 0.046 | 2.383 | 0.020 |
|  |  | β_2_ | -0.350 | -1.447 | 0.153 |
|  |  | β_3_ | -0.084 | -3.251 | 0.002 |
|  | >14 years old | β_1_ | 0.001 | 2.113 | 0.039 |
|  |  | β_2_ | 0.000 | -0.029 | 0.977 |
|  |  | β_3_ | -0.003 | -3.737 | <0.001 |


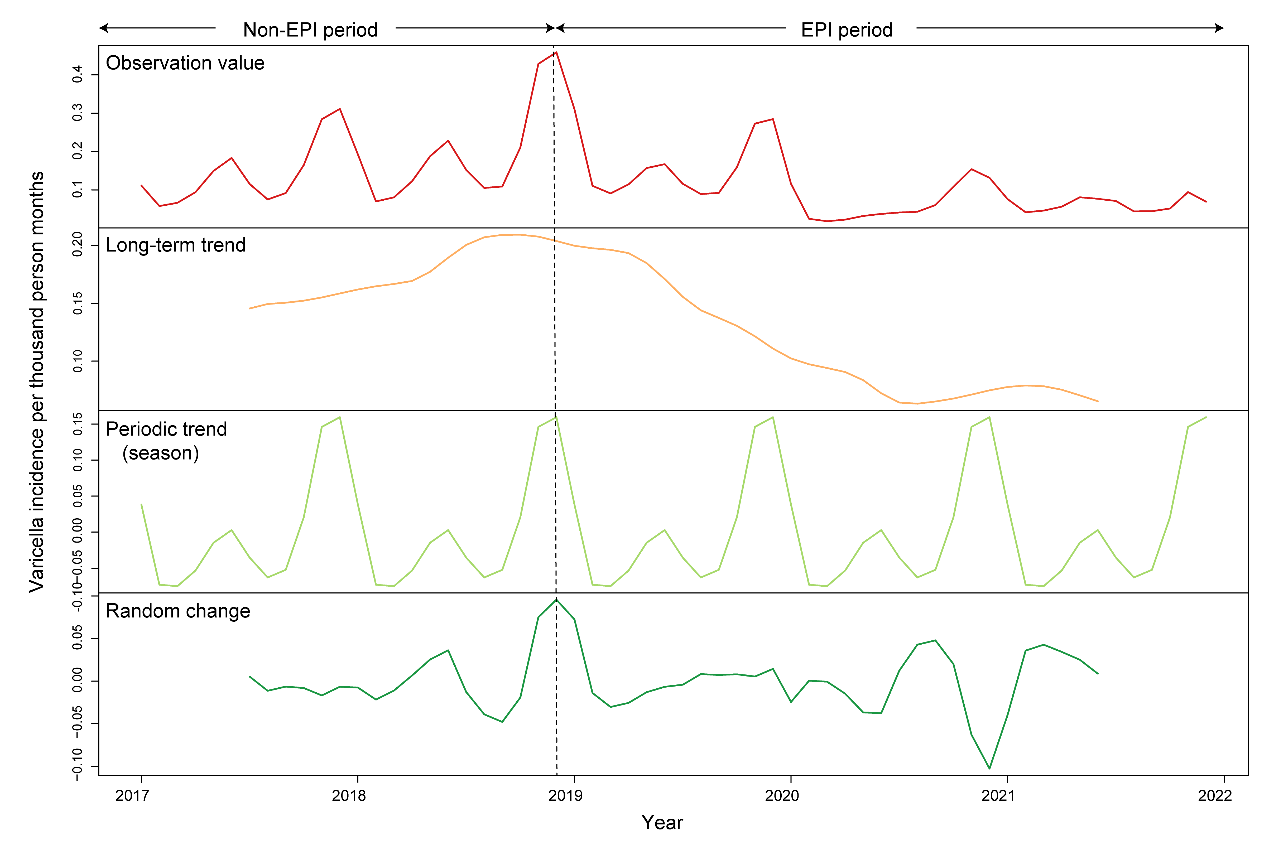


**Figure S3. Decomposing of varicella incidence from 2017 to 2021**
